# Latent fingerprint development ability of spinel copper aluminate (CuAl_2_O_4_) nanoparticles

**DOI:** 10.3389/fchem.2026.1830486

**Published:** 2026-06-10

**Authors:** Rameen Fatima, Sammia Shahid, Sana Mansoor, Momna Qayyum, Komal Aroosh, Mohsin Javed, Mah Rukh, Mohammed Abohashrh, Fadi Jaber, Shahid Iqbal, Sajid Mahmood, Abeer Ahmed Alghamdi, Shaimaa A. M. Abdelmohsen

**Affiliations:** 1 Department of Chemistry, School of Science, University of Management and Technology, Lahore, Pakistan; 2 Department of Basic Medical Sciences, College of Applied Medical Sciences, King Khalid University, Abha, Saudi Arabia; 3 Department of Biomedical Engineering, Ajman University, Ajman, United Arab Emirates; 4 Center of Medical and Bio-Allied Health Sciences Research, Ajman University, Ajman, United Arab Emirates; 5 Department of Chemical and Environmental Engineering, University of Nottingham Ningbo China, Ningbo, China; 6 Low Dimensional Materials Research Center at Khazar University, Baku, Azerbaijan; 7 Department of Physics, College of Science, Princess Nourah bint Abdulrahman University, Riyadh, Saudi Arabia

**Keywords:** copper aluminate, heterogeneous gels, latent fingerprints (LFPs), sol-gel synthesis, spinel CuA2O4

## Abstract

Fingerprints are still one of the most effective means of forensic detection but degradation during the recovery process leads to a loss of contrast between ridge and background, making them difficult to visualize. In this study spinel copper aluminate (CuAl_2_O_4_) nanoparticles were synthesized by a facile and cost effective sol-gel method and evaluated for latent fingerprints (LFPs) development on both porous (paper, ceramic, wood) and non-porous (plastic, steel, glass) substrates. Structural characterizations revealed an average crystallite size of 24.5 nm that was found to be smaller than the average particle size of 36.9 nm confirming that the particles consists of nanoscale crystalline domains with some agglomeration. The developed fingerprint exhibited substrate-dependent contrast with normalized contrast ratios increasing from ∼0.30 (paper) to ∼0.60 (glass) under white light and further enhanced to ∼0.74 under UV excitation. UV-assisted visualization enabled improved ridge clarity and facilitated the observation of Level 1 and Level 2 details, with partial enhancement towards finer features. Aging studies demonstrated that identifiable ridge patterns were retained up to 30 days, with improved visibility under UV illumination compared to white light conditions. These findings demonstrate that CuAl_2_O_4_ nanoparticles provide simple, cost-effective, and reliable approach for fingerprint development, combining surface adhesion and luminescent enhancement for improved forensic applicability.

## 1 Introduction

Nano forensics replaces the complicated and bulky devices with nanotechnology to improve the reliability and precision of identifying undetectable evidence in crime scene investigations (Ullah et al., 2024). It entails developing sophisticated instruments for applications including explosive screening, ammunition residue analysis, fingerprint visualization, illegal drug examination, and bodily fluid detection (Wang et al., 2023). It offers improved methods for real-time criminal prosecutions, such as the nano-imaging, nano-manipulators, and nano-sensors (Dahiya et al., 2022). When a person touches an object, they leave their unique fingerprint pattern, which do not alter over the time. These patterns give crucial information that can aid in the detection of victims and suspects (Singh and Minj, 2024). Sweating residue from friction ridge skin pores create fingerprints. This process is mediated by three glands, namely, the eccrine, apocrine, and sebaceous. Eccrine glands produce clearer and colorless sweat which is composed of approximately 99% of water, and 0.5% organic and 0.5% inorganic substances. Eccrine sweat is known to have different constituents which include amino acids, proteins, choline, creatinine, lactic acid, carbohydrates, urea and uric acid. Conversely, the sebaceous sweat is composed of squalene, glycerides, wax, sterol esters and fatty acids (Bumbrah et al., 2016; Dhall and Kapoor, 2016). These fingerprint impressions, also known as the latent fingerprints (LFPs), are often not visible to the naked eye and require special methods of the visualization (Yuan et al., 2021). A variety of procedures, including physical, chemical, and/or optical techniques, are commonly utilized either individually or in a predetermined sequence for LFPs development on various surfaces (Grover et al., 2024).

Physical methods utilize powdered compositions to physically interact with latent fingerprint residues on the substrates. Chemical methods involves the transformation of some particular element in sweat to colored derivative (Bu et al., 2023). Optical technologies are non-destructive in nature, and they utilize electromagnetic radiation to reveal LFPs. The choice of development approach depends on the color, condition, nature and texture of the surface on which the fingermark has been deposited (Bumbrah et al., 2019). Powder dusting is simple, quick, widely applied, and efficient way to develop LFPs on dry, non-porous substrates. Finely powdered formulation is applied to fingerprint residuals, and excess powder is removed by brushing and blowing (Prabakaran and Pillay, 2020). The powder’s adherence qualities are determined by particle shape and size, with small, fine particles sticking more strongly than that of the large, coarse particles (Hopkins et al., 2023). There is no single powder composition that can detect LFPs on all of the surfaces. The powder dusting approach is utilized for the majority of fingerprint identifications worldwide; therefore, even the minor of enhancements due to variations in powder composition could give substantial functional advantages (Vadivel et al., 2021).

Traditional fingerprint powders were commonly employed to develop LFPs on variety of surfaces. Different nanomaterials, such as metal oxides, carbon nanostructures, and silica nanoparticles have been investigated. For example, Bavage et al. has developed silica (SiO_2_) nanoparticles (NPs) from biomass. These eco-friendly SiO_2_ NPs have shown enhanced adhesion and surface activity for the successful visualization of fingerprints on various surfaces (Bavage et al., 2025). However, many of these systems rely primarily on physical contrasts under white light and may exhibit limitations in background suppression particularly on complex or low-contrast surfaces. Metallic powders typically have longer shelf life than organic powders. Lanthanides with luminous powders have been exploited to visualize LFPs on the multicolored substrates (Darshan et al., 2016). However, they are rarely employed owing to their toxic characteristics and complicated deployment method (Farrukh, 2015). Nanopowder-based materials have recently been employed for LFPs identification instead of traditional fingerprint powders due to their great adhesion, precision, and reduced background staining as well as contamination (Bumbrah et al., 2022). For instance, Dubey et al. developed hexagonal CuO nanoparticles for developing fingerprints with improved contrast and visibility (Dubey et al., 2025). Mukherjee et al. reported the synthesis of Cu^2+^ incorporated Mg_2_AlO_4_ nanomaterial for LFPs development on smooth surfaces (Mukherjee et al., 2020). In another study, Cu-doped ZnO nanoparticles were used as labeling agent by Naik et al. to visualize LFPs on diverse substrates using the powder dusting approach (Naik et al., 2021).

Aluminate-based nanopowders have sparked considerable interest among material scientists as labeling agents in LFPs development due to their exceptional characteristics, including high melting points, hardness, mechanical strength, chemical and thermal stability, wide energy band gap, and high electrical resistivity (Doroftei and Leontie, 2021; Ragupathi et al., 2014). These attributes of aluminates make them ideal candidate for diverse applications, such as sensors, biomedical devices, and display technologies. Structurally the aluminate spinel-type mixed oxides (AB_2_O_4_), constitute cubic spinel framework where A^2+^ (Ca, Mg, Cu, Ni, Fe, Zn, Sr, etc.) occupies the tetrahedral positions and B (Al^3+^) ions resides in octahedral sites, although B can also be distributed between both of the sides (Spiridigliozzi et al., 2023). Various aluminate spinels like NiAl_2_O_4,_ ZnAl_2_O_4_, SrAl_2_O_4_, CuAl_2_O_4_, etc., have extensively been studied for that of the functional applications. Moreover, while several reported materials demonstrate sufficient adhesion properties, the incorporation of luminescent properties for the improved visibility is still relatively underdeveloped or involve complicated synthesis. The quest for improved adhesion, compatibility with different substrates and the ability to enhance optical properties is an ongoing challenge in the design of new materials for fingerprint detection. In this regard, spinel-type CuAl_2_O_4_ nanoparticles is an attractive alternative owing to that of its inherent stability, nano-scale size and photoluminescence (Karsharma et al., 2025). In this context spinel-type CuAl_2_O_4_ nanoparticles presents promising alternative due to their inherent structural stability, nanoscale morphology, and photoluminescent properties (Srinath et al., 2024). The combination of surface interaction capability with that of the UV responsive emission offers the opportunity to improve ridge contrast and reduce background interference. Therefore, the present study focuses on the synthesis and evaluation of CuAl_2_O_4_ nanoparticles as potential material for LFPs detection across different substrates and aging conditions.

In this work sol-gel method was used for the fabrication of spinel CuAl_2_O_4_ nanoparticles as an effective luminescent tool for developing latent fingerprints across porous and non-porous substrates, which are challenging for conventional techniques, offering consistent and persistent visibility for forensic applications. The sol-gel method facilitated simple and cost-effective synthesis of these uniform nanoparticle (Wang et al., 2021), and were characterized using XRD, FTIR, SEM, and EDX. The luminescent characteristic of the material was also examined under UV light for enhanced contrast and ridge details. Additionally, the durability of the material also investigated by conducting aging studies after regular intervals of time. This work offers safer, more effective alternative to conventional methods, with better stability, reduced hazard, and improved application on various surfaces. It validates the physical durability and operational readiness of CuAl_2_O_4_ nanoparticles, providing valuable insights to nano-forensics by demonstrating the reliability of the material for future crime scene applications.

## 2 Experimental

### 2.1 Chemicals

All of the materials were of analytical grade and were used without any further purification. These included the copper nitrate trihydrate (Cu(NO_3_)_2_.3H_2_O-Sigma Aldrich 99%), aluminum sulfate (Al_2_(SO_4_)_3_-Sigma Aldrich 99.9%), Anhydrous citric acid (HOC(COOH)(CH_2_COOH)_2_-Sigma Aldrich 99.5%), aqueous ammonia (25%, Sigma Aldrich), hydrochloric acid (HCl-ACS reagent 37%). The deionized water was used throughout the experiment.

### 2.2 Instrumentation

The powdered sample was examined using Shimadzu XRD-6000 diffractometer with CuKα (0.154 nm) radiation at 30mA and 40 kV. For morphological characterization and elemental analysis, Hitachi S-400 scanning electron microscopy (SEM) equipped with an Energy Dispersive X-ray (EDX) spectrometer was employed at low voltage of 1.2–1.5 kV for 30 s. Fourier Transform infrared spectroscopy (FTIR) was conducted with PerkinElmer Spotlight 200 instrument, using the Attenuated Total Reflectance (ATR) approach, to evaluate the infrared (IR) spectra. The Photoluminescence (PL) emission spectrum was recorded using Horiba FluoroMax Spectrofluorometer (HORIBA Ltd. Japan) employing a photomultiplier tube (PMT) detector.

### 2.3 Synthesis of CuAl_2_O_4_ nanoparticles

**FIGURE 1:**
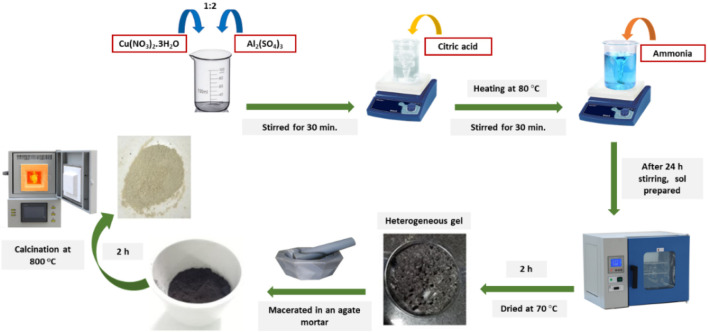
Schematic illustration for the synthesis of CuAl_2_O_4_ nanoparticles.

CuAl_2_O_4_ nanoparticles were synthesized using sol-gel method. Initially, 0.04 mol of Cu(NO_3_)_2_·3H_2_O and 0.08 mol of Al_2_(SO_4_)_3_ were dissolved in 100 mL of the deionized water to form solution A, with molar ratio of Cu^2+^ to Al^3+^ of 1:2. Separately, solution B was prepared by dissolving the 0.24 mol of anhydrous citric acid in the 100 mL of deionized water. Solution B was then dropped into solution A with constant stirring at the 30 °C and the mixture was heated gradually to 80 °C to obtain bluish-green solution. The mixture was stirred for another half hour. Then the pH was adjusted to two by adding 25% of the aqueous ammonia. If the pH exceeds the desired level, then HCl was used to keep the pH balanced. The mixture was stirred with the help of magnetic stirrer during 24-h period to attain dark-blue solution. The sol was then heated at 100 °C, for about 2 h to produce dark, brownish black, honeycomb shaped, heterogeneous gel. The gel was ground into fine powder and finally calcined at the 800 °C for 2 h in muffle furnace to produce spinel CuAl_2_O_4_ nanoparticles. Figure 1 displays synthetic scheme for the CuAl_2_O_4_ nanoparticles.

### 2.4 LFPs development and evaluation

Prior to fingerprint deposition the substrates were cleaned to remove dust and surface contaminants. Non-porous substrates such as the glass, plastic, and steel were gently wiped with ethanol and allowed to dry under the ambient conditions. Porous substrates including the paper, wood, ceramic were used as received without additional treatment. This approach ensured consistent surface condition for that of the comparative analysis. The CuAl_2_O_4_ NPs nanopowder was carefully ground and then dusted onto LFPs, placed on the substrates, using the magnetic brush. A small quantity of nanopowder was used to ensure the even coverage on the fingerprint ridges and the excess powder was carefully brushed off to avoid any of the powder residues on the background while enhancing the ridge clarity. Experiments were conducted at the room temperature (25 °C ± 5) and relative humidity (50 ± 5) in laboratory environment, and identical experimental conditions were used throughout the experiments. Fingerprint development was evaluated on both of the porous (paper, ceramic, wood) and non-porous (plastic, steel, glass) surfaces to determine effectiveness on the range of surfaces. The fingerprints were captured using high resolution mobile phone camera.

For the luminescence aided detection, the developed fingerprints were exposed under UV lamp (365 nm) to obtain better visualization of the ridge details owing to that of the photoluminescent properties of the material. The images were captured for the further analysis. In order to assess the stability and applicability of developed material, a study of developed fingerprints over time was carried out. The samples were kept in the ambient laboratory conditions and were analyzed after 10, 20 and 30 days. The samples were examined under both of the white light and UV light in order to determine the preservation of ridge detail. The reproducibility of the fingerprint development was ensured by conducting the experiments on several different substrates under the same conditions. The ridge development pattern was consistent throughout the repeated trials, ensuring the method’s reproducibility.

### 2.5 Image-based contrast analysis

The effectiveness of the fingerprint development was assessed by measuring the normalized contrast through the ImageJ. Firstly, all of the images were converted to the 8-bit grayscale (intensity range: 0-255) to allow for intensity-based evaluation. Rectangular regions of interest (ROI) were manually chosen from representative ridge regions and background regions without fingerprint residue using fixed-size rectangle tool. To avoid any of the possible bias three separate measurements from both ridge and background regions were taken for each sample and their average intensity values was used for calculations. The contrast was evaluated by means of normalized intensity difference as per the equation given below Equation 1:

$$Contrast=\frac{I_{background}-I_{ridge}}{I_{background}}$$ (1)

Where I_ridge_ and I_background_ represent the mean grayscale intensities of the fingerprint ridge and the surrounding substrate respectively. This formulation provide dimensionless metric (ranging from 0 to 1) that reflects the degree of ridge-background differentiation with the higher values indicating improved visibility (Kellman et al., 2014).

## 3 Results and discussion

### 3.1 XRD analysis

**FIGURE 2:**
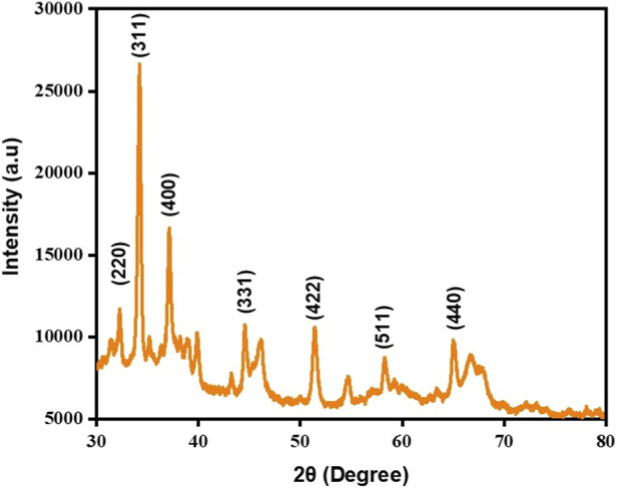
XRD pattern of CuAl_2_O_4_ nanoparticles.

The crystalline structure of the synthesized copper aluminate nanoparticles was examined using XRD, as shown in Figure 2. The XRD pattern of CuAl_2_O_4_ NPs demonstrated well-defined peaks corresponding to spinel phases of CuAl_2_O_4_. It exhibited characteristic diffraction peaks at the 2θ values of approximately 31.8^ο^, 34.1^ο^, 37.1^ο^, 44.5^ο^, 51.4^ο^, 58.2^ο^, and 64.9^ο^, corresponding to the crystallographic planes indexed as (220), (311), (400), (422), (511), and (440). This XRD pattern is consistent with the JCPDS card no. 33-0448 (Tan et al., 2021). The average crystallite size was estimated using the Scherrer equation given below (Equation 2):

$$D=\frac{k\lambda }{\beta \cos\theta }$$ (2)

Where D is the crystallite size, λ is the wavelength, k is the Scherrer constant, β the full width at half maximum (FWHM), and θ is the Bragg angle. It was calculated to be 24.5 nm for the prepared CuAl_2_O_4_ NPs. Salavati-Niasari et al. reported the crystallite size of 28 nm for CuAl_2_O_4_ NPs prepared via sol-gel method (Salavati-Niasari et al., 2009). The obtained crystallite size and the phase purity are in good agreement with that of the previously reported CuAl_2_O_4_ nanoparticles synthesized through the similar methods (Kwak et al., 2012) confirming the structural integrity and nanoscale characteristics of prepared nanomaterial.

### 3.2 FTIR analysis

**FIGURE 3:**
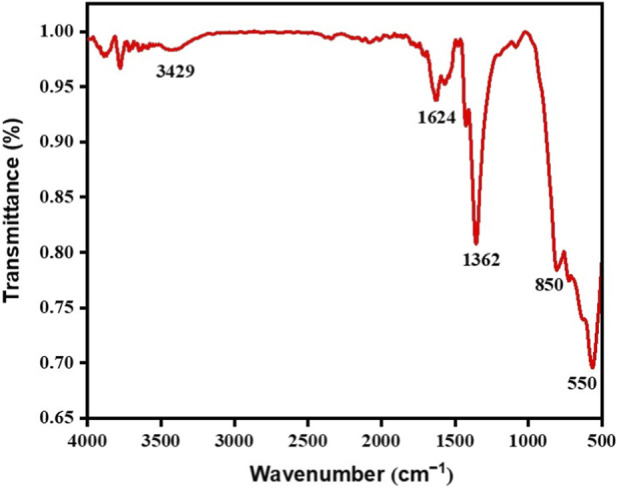
FTIR spectrum of the CuAl_2_O_4_ nanoparticles.

FTIR spectrum of CuAl_2_O_4_ nanoparticles confirm the spinel structure, as demonstrated in Figure 3. The characteristic bands observed in low-frequency range 500–900 cm^-1^ are related to the metal-oxygen stretching vibrations. The peaks at wavenumbers 550 cm^-1^ and 850 cm^-1^ are related to that of the Cu-O and Al-O vibrations respectively, confirming the formation of the CuAl_2_O_4_ lattice (Gholami and Maddahfar, 2016). A broad band observed at the 3429 cm^-1^ and peak near 1624 cm^-1^, corresponds to the OH stretching and H-O-H bending vibrations, respectively, indicating the presence of the adsorbed moisture on the nanoparticle surface (Qayyum et al., 2025). Additionally, the peak at 1362 cm^-1^ was also observed, which may be attributable to trace carbonate species or surface residues, likely originating from atmospheric adsorption or minor remnants of precursor decomposition employed in sol-gel process (Tangcharoen et al., 2018). The persistence of these bands despite calcination at the 800 °C, further indicate that these species are mainly linked to surface interactions rather than that of the bulk structural contribution. This highlight their surface-associated functionality while the overall spinel framework remains structurally stable.

### 3.3 SEM analysis

**FIGURE 4:**
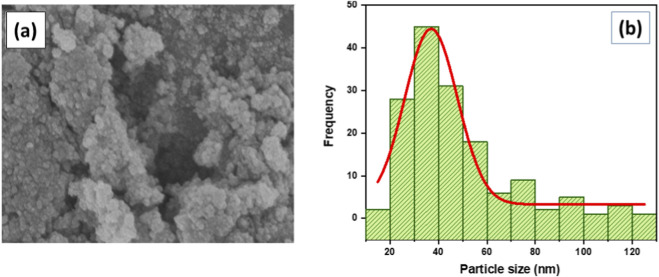
**(a)** SEM morphology and **(b)** the corresponding size distribution histogram of spinel CuAl_2_O_4_ nanoparticles.

The morphological analysis of the synthesized CuAl_2_O_4_ nanoparticles is displayed in Figure 4. Agglomerated clusters of nanoscale particles with irregular to semi-spherical morphology is revealed by the SEM image, as can be observed in Figure 4a. These aggregates consist of fine primary grains that are closely packed, resulting in relatively homogenous surface distribution. This morphology is in alignment with copper aluminate nanostructures that have previously been reported (Kumar et al., 2012; Talebi, 2016).

The particle size was measured to offer quantitative insights (Figure 4b). With average particle size centered around 36.9 nm, the histogram distribution curve reveal that most of the particles are dispersed between the 30–60 nm. Partial agglomeration of nanoparticles is suggested by moderate tail that extend toward the larger diameters (∼120 nm), which is frequently seen in spinel-type oxide systems. Interestingly, the SEM particle size is slightly larger than that of the XRD crystallite size (∼24 nm), indicating that measured particles comprised of multiple crystallites resulting in the agglomerated structure. This is commonly observed for nanomaterials synthesized through sol-gel route, where primary nanocrystals tend to aggregate during the calcination process (Srinath et al., 2023). The aggregated morphology and the nanoscale particle size increase the effective surface area, which improve the adhesion of the nanoparticles with that of the fingerprint residue (Qayyum et al., 2024).

### 3.4 EDX analysis

**FIGURE 5:**
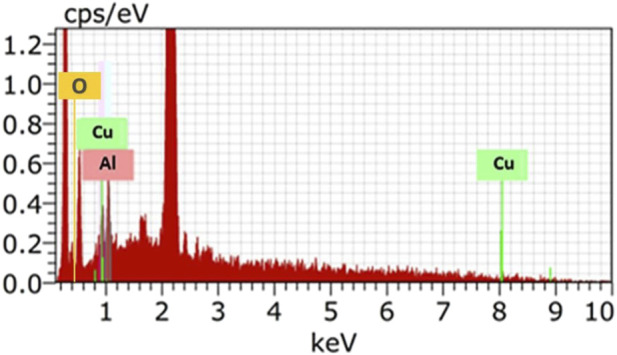
EDX analysis of CuAl_2_O_4_ nanoparticles.

The elemental composition of the sample was confirmed by EDX analysis (Bashir et al., 2025). The EDX spectrum of the synthesized CuAl_2_O_4_ nanoparticles confirmed the presence of copper (Cu), aluminum (Al), and oxygen (O) as demonstrated in Figure 5. This confirmed efficient synthesis of spinel CuAl_2_O_4_ nanoparticles. Additionally, the corresponding elemental compositions obtained from the EDX analysis is presented in Table 1. The observed elemental ratios are in reasonable agreement with the expected stoichiometry of CuAl_2_O_4_ nanoparticles validating the successful formation of the spinel phase. The absence of additional elemental signals further confirms the purity of the synthesized nanoparticles.

**TABLE 1:** Quantitative elemental composition of CuAl_2_O_4_ nanoparticles obtained from EDX analysis.

Elements	Atomic %	Weight %
Cu	14.7	31.5
Al	24.8	26.1
O	60.5	42.4

### 3.5 Fingerprint development on porous and non-porous surfaces

**FIGURE 6:**
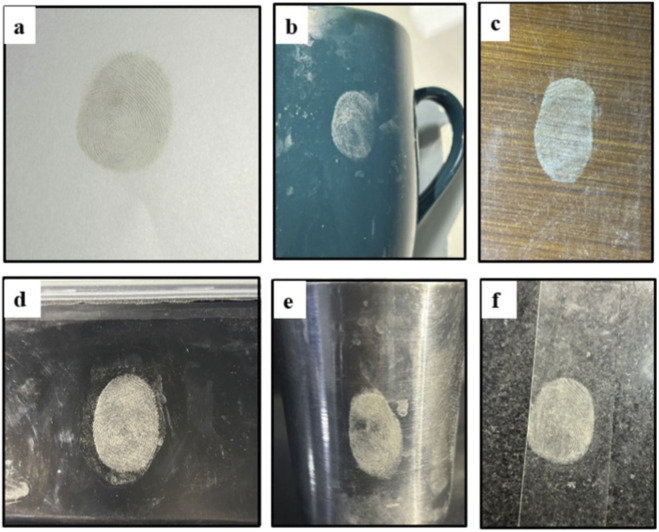
Fingerprint development under the white light on porous surfaces: paper **(a)**, ceramic **(b)**, and wood **(c)**, as well as non-porous surfaces: plastic **(d)**, steel **(e)**, and glass slide **(f)**.

In order to determine the efficiency of spinel CuAl_2_O_4_ nanoparticles in developing fingerprints on diverse surfaces, both of the porous and the non-porous substrates were tested under the white light as indicated in Figure 6. On the porous surfaces, such as paper, ceramic, and wood (Figures 6a–c), the powder sticks firmly to that of the fingerprint residues, leaving distinct ridge patterns. In the same manner, on non-porous surfaces like plastic, steel and glass slides, clear fingerprint impression was made with good contrast between the background and the ridges as shown in Figures 6d–f. These findings illustrate the applicability and capability of spinel CuAl_2_O_4_ in developing LFPs on the diverse surfaces, which underscore its possible utility in the forensic investigation.

Based on the initial comparative evaluation across different substrates, glass surface exhibited the highest clarity and contrast. Therefore, it was further selected for detailed UV-assisted analysis to ensure consistent and reliable assessment of the material’s photoluminescent performance.

### 3.6 Fingerprint visualization under UV light

**FIGURE 7:**
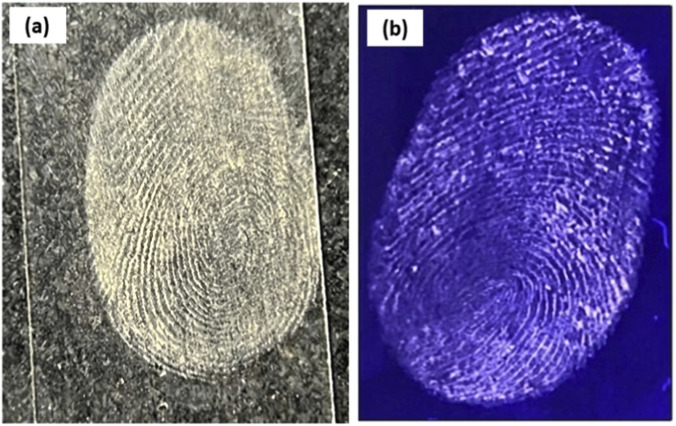
Developed a fingerprint on glass slide under white **(a)** and UV light **(b)**.

By leveraging the photoluminescent properties of CuAl_2_O_4_ NPs further enhancement of visualization of LFPs were achieved under UV illumination. As illustrated in Figure 7 the deposited material exhibited visible emission leading to increased ridge-to-background contrast under excitation. This luminescent response significantly improved the clarity of ridge pattern compared to visible light observation, particularly on non-porous substrates. While the Level 3 (sweat pores) characteristics were not resolved distinctly, Level 1 (overall ridge flow) and the Level 2 minutiae details (such as bifurcations and ridge ends) were easily discernible. The improvement under UV light exhibit the ability of the CuAl_2_O_4_ NPs combining physical powder adhesion with optical contrast improvement, thereby enabling more effective fingerprint visualization under the challenging conditions.

### 3.7 PL analysis

**FIGURE 8:**
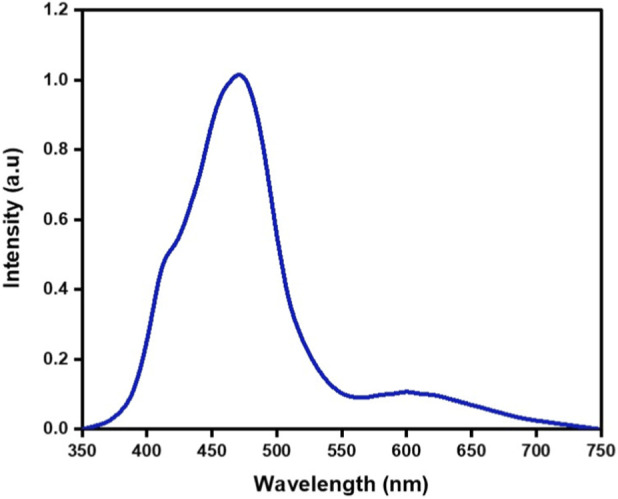
PL emission spectrum of CuAl_2_O_4_ nanoparticles.

The photoluminescence (PL) properties of the synthesized CuAl_2_O_4_ NPs were examined by recording the spectra in the wavelength range of 350–750 nm under excitation at the 325 nm at room temperature (Figure 8). The emission spectrum exhibited broad and intense band centered in the blue region of ∼460–480 nm which are consistent with the reported luminescence behavior of spinel CuAl_2_O_4_ nanomaterials (Chitra et al., 2024). The broad profile indicates the involvement of both of the intrinsic transitions and defect-related or surface-associated electronic states. In addition to the dominant blue emission, weaker secondary emission features were observed in the higher wavelength region of ∼580–620 nm, which suggest the presence of additional localized energy states within the lattice, commonly associated with structural imperfections or surface defects in the nanocrystalline systems. The observed luminescence behavior is therefore consistent with the nanocrystalline and slightly aggregated nature of the material as indicated by that of the XRD and SEM analysis which can introduce the defect sites contributing to the emission profile.

The effectiveness of the CuAl_2_O_4_ nanoparticles in the development of LFPs could be ascribed to the synergy of surface interactions, structural features, and optical properties. The complex mixture of organic components found in latent prints such as the lipids, amino acids, and moisture, interacts physically with the powder. This govern the adhesion of the nanoparticles with that of fingerprint residues. The large surface area from the nanoscale particle size maximize contact with ridge residues and facilitate selective adhesion while reducing the deposition on background surface (Sahu and Jain, 2025). Moreover, the morphology of the synthesized particles comprises of fine grains with slight aggregation which can effectively cover the ridge patterns without remarkably masking final structural details. Improved adhesion behavior may be supported by that of the surface-associated functions as revealed by the FTIR analysis, which may also contribute to the interaction with fingerprint constituents.

CuAl_2_O_4_ NPs' luminescence behavior under the UV light is associated with electronic transitions in the spinel lattice as well as defect-related states such the oxygen frequencies or the lattice distortions introduced during synthesis and calcination (Tangcharoen et al., 2019). Emission in the visible spectrum can result from these defect states acting as luminescent centers. By intensifying the signal from the that of the deposited nanoparticles and reducing background interference under UV stimulation, this photoluminescent response improve ridge contrast as well as the visualization of fingerprint features (Pinna et al., 2023). Overall the combined influence of the nanoscale size, surface characteristics, and luminescence properties enable effective ridge development and enhanced fingerprint detectability across the different substrates.

### 3.8 Contrast analysis of fingerprint visualization

The calculated contrast values demonstrated clear dependence on substrate type and illumination conditions, while also reflecting the performance of developed CuAl_2_O_4_ material. Relatively low contrast of ∼0.30 was observed for the fingerprint developed on paper under white light. This can be ascribed to the rough and porous nature of the substrate which thus leading to the increased background interference and the reduced effectiveness of ridge differentiation. Conversely, improved contrast of ∼0.60 was demonstrated by fingerprints on the glass substrate under the white light, suggesting the better compatibility of CuAl_2_O_4_ NPs with that of the smoother non-porous surface. Under UV light, further increase in contrast of about 0.74 was seen. This can be explained by the developed material’s photoluminescent response, which increase ridge signal strength and suppress background contribution, improving visibility.

Overall the progressive enhancement in contrast from the paper to that of the glass under UV excitation confirm that fingerprint visualization is governed not only by substrate properties but also by the functional performance of the developed material. The ability of CuAl_2_O_4_ NPs to reduce background interference and amplify ridge specific signals demonstrate its effectiveness for reliable fingerprint detection across the varying conditions.

### 3.9 Fingerprint aging study

**FIGURE 9:**
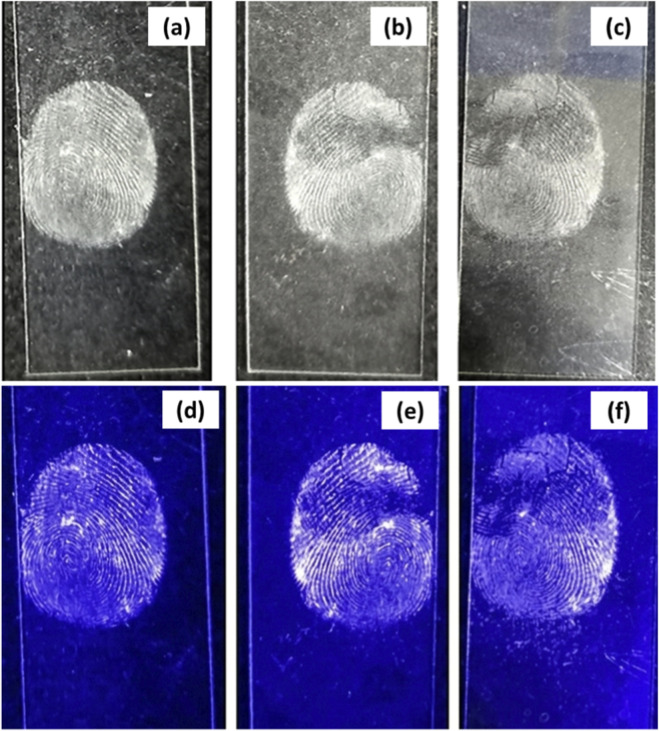
Aging behavior of developed fingerprints under white and UV illumination after 10 **(a,d)**, 20 **(b,e)**, and 30 **(c,f)** days.

The effectiveness of CuAl_2_O_4_ NPs in the LFPs development was evaluated over the aging periods of 10, 20 and, 30 days as shown in Figure 9. Gradual reductions in fingerprint clarity was absorbed with increasing aging time which is consistent with that of the natural degradation of fingerprint residues. Over the time volatile components such as moisture evaporate, while lipid and organic constituent undergoes oxidation and redistribution, leading to reduced adhesion sites for that of the powder particles (Cadd et al., 2015). Despite of these changes developed fingerprints retained identifiable ridge characteristics even after the 30 days demonstrating the ability of the nanoparticles to interact with residual components of aged prints. Compared to the white light circumstances, better visibility was seen under UV light (Figures 9d–f), suggesting that the material’s luminescent response compensate for aged samples' lowered physical contrast. These findings implies that CuAl_2_O_4_ NPs offer reliable and consistent performance for detecting of aged fingerprints while preserving adequate ridge visibility for forensic examination over the long periods of time.

### 3.10 Ridge clarity evaluation on different substrates

Using semi-quantitative ridge clarity scoring system (1-5) adopted from the established literature (Verma et al., 2022) which is based on the ridge continuity, pattern visibility along with the degree of background interference, quality of the developed LFPs was further assessed. The scoring criteria allowed a systematic comparison of different substrates, lighting conditions and aging times. The development and visualization of fingerprint exhibited pronounced dependence on substrate characteristics under the white light as summarized in Table 2. Porous and irregular substrates like paper, wood and steel showed the lower clarity score (2-3) which can be attributed to uneven powder adhesion, surface roughness and increased background interference. Alternatively, smoother and non-porous substrates, such as the glass showed improved definition of ridges, achieving the clarity score of four under white light conditions.

**TABLE 2:** Clarity score of LFPs on different substrates under white and UV light, including the aging conditions.

Substrate	Illumination	Aging time (days)	Clarity score	Quality level
Paper	White	0	2	Poor
Ceramic (mug)	White	0	2	Poor
Wood	White	0	3	Moderate
Plastic	White	0	3	Moderate
Steel	White	0	2	Poor
Glass Slide	White	0	4	Good
Glass Slide	UV	0	5	Excellent
Glass slide	White	10	3	Moderate
Glass slide	UV	10	4	Good
Glass slide	White	20	3	Moderate
Glass slide	UV	20	4	Good
Glass slide	White	30	2	Poor
Glass slide	UV	30	3	Moderate

The significant enhancement in fingerprint visualization was observed when the substrates was exposed to the UV light. The fresh fingerprints that was developed on glass substrate showed highest clarity score (5) suggesting good ridge continuity, contrast, and least background interference. This improvement is attributed to the photoluminescent response of the material that enhance ridge-specific signal while reducing substrate-related noise. Aging studies conducted over the 10, 20 and 30 days revealed gradual decrease in clarity under the white light conditions with scores decreasing from moderate (3) to the poor (2), likely due to degradation and diffusion of residue over the time. However, under UV illumination the fingerprints retained comparatively higher visibility with clarity scores between three and four even after the 30 days. This demonstrate that UV-assisted visualization improve the detectability of aged fingerprints and highlight the robustness of the developed material for long-term forensic applications.

### 3.11 Comparative studies

A comparative analysis was performed to evaluate the performance of the CuAl_2_O_4_ nanoparticles against the previously reported fingerprint development materials and is summarized in Table 3. The comparison was based on key parameters including synthesis method, substrate compatibility, particle size illumination conditions, ridge detail sensitivity, and aging stability.

**TABLE 3:** The effectiveness of various materials for LFPs development.

Material	Synthesis method	Substrates	Average particle size	Illumination	Contrast	Aging	References
Mesoporous Silica NPs	Sol-gel	Porous, Non-porous	100–150 nm	UV torch (365 nm)	Level 1/2	<30 days	Bhati et al. (2023)
C-dots@TiO_2_	Hydrothermal method	Non-Porous	core–shell (∼500 nm shell); C-dots 2–5 nm	White light	Level 1/2/3	7 days	Yadav et al. (2018)
ZnO NPs	Commercial procurement	Non-porous	38 nm	White light	Level 1/2	14 days (glass slide)	Mode-on et al. (2025)
CuO NPs	Green sonochemical synthesis	Non-porous	500–900 nm	White light	Level 1/2	---	Bhagat et al. (2021)
TiO_2_:Ce^3+^ nanophosphors	Solution combustion method	Porous, Non-porous	9–30 nm	White light	Level 1/2/3	21 days	Babu et al. (2018)
CuO nanosheets	Hydrothermal method	Porous, Non-porous	----	White light	Level 1/2	7 days	Atri et al. (2024)
CuAl_2_O_4_ NPs	Sol-gel	Porous, Non-porous	36.9 nm	White light/UV lamp (365 nm)	Level 1/2	30 days	This work

Most nanoparticle based system demonstrate limited sensitivity typically revealing the Level 1 and Level 2 ridge details with occasional observation of the Level 3 features under optimized conditions. In agreement with these reports, the CuAl_2_O_4_ NPs developed in this study enables clear visualization of Level 1 ridge pattern along with distinct Level 2 features such as ridge endings and bifurcations across both of the porous and non-porous substrates. While Level 3 details were not distinctly resolved the material exhibits consistent and reliable performance in revealing essential ridge characteristics required for fingerprint identification. Notably compared to several reported systems with limited aging stability, the developed fingerprint in this study retain identifiable rich structures for up to 30 days under ambient conditions. Additionally, the material demonstrates effective performance under both white and UV illumination providing enhanced contrast and improved detectability particularly on smooth substrates, without the integration of any luminescent material. These results indicate that although the sensitivity level is comparable to existing net nanoparticle based system CuAl_2_O_4_ NPs offers improved practical applicability through their combined stability substrate versatility, and dual-mode visualization capability.

## 4 Conclusion and future perspectives

In the current research spinel CuAl_2_O_4_ nanoparticles were successfully prepared by simple sol-gel process and proved to be effective for developing LFPs on both porous and non-porous surfaces. The synthesized nanoparticles showed good binding properties with fingerprint residues allowing ridge pattern to be clearly discerned with consistent Level 1 and Level 2 detail. The luminescent property of the nanoparticle under UV excitation offers better contrast compared to the white light resulting in improved visibility of fingerprints particularly on non-porous surfaces. Photoluminescence studies further confirmed intrinsic optical response of CuAl_2_O_4_ nanoparticles. While the aging analysis also indicated that identifiable ridge pattern remained detectable for up to the 30 days suggesting good fingerprint stability. The overall performance of CuAl_2_O_4_ nanoparticles can be attributed to the combined effect of the nanoscale morphology, surface-associated interaction and photoluminescence properties collectively supporting its potential as promising and scalable material for forensic fingerprint development. In addition to the present findings, the study also highlights several directions for future advancements. Quantitative analysis of detection sensitivity in different experimental settings such as humidity and temperature could further improve the practical forensic reliability. Also, surface modification strategies might be investigated to improve the selective binding to fingerprint residues and improve visualization efficiency on challenging substrates. Furthermore, combining CuAl_2_O_4_ NPs with portable or the real-time imaging platforms may broaden their applicability in field-based forensic investigation. Extended validation on more complex substrates and under the diverse illumination environments would additionally contribute towards strengthening real-world applicability and long-term operational performance.

## Data Availability

The raw data supporting the conclusions of this article will be made available by the authors, without undue reservation.
